# Systemic Arterial Hypertension in Patients Exposed to Cesium-137 in Goiânia-GO: Prevalence Study

**DOI:** 10.5935/abc.20170062

**Published:** 2017-06

**Authors:** José Victor Rabelo Rodrigues, Murillo Macêdo Pinto, Roberto Miller Pires Figueredo, Helen de Lima, Rafael Souto, Sylvana de Castro Sacchetim

**Affiliations:** 1 Centro Universitário de Anápolis - UniEVANGÉLICA, GO - Brazil; 2 Secretaria da Saúde do Estado de Goiás, GO - Brazil

**Keywords:** Hypertension, Cesium, Cesium, Radioisotopos, Cardiovascular Diseases

## Abstract

**Background:**

Systemic Arterial Hypertension (SAH) in the Brazilian population, in populations not exposed to Césio-137, presents a prevalence of 28% nationwide. However, in the group of radioactivity victims, these values are unknown.

**Objective:**

To analyze the prevalence of hypertension in patients exposed to Cesium-137 in Goiânia, enrolled in the Sistema de Monitoramento dos Radioacidentados (SISRAD) (Radioactivtity Victims Monitoring System) of the Centro de Assistência aos Radioacidentados (C.A.R.A) (Assistence Center for Radioactivity Victims).

**Methods:**

This is a descriptive, observational cross-sectional epidemiological study carried out in Goiânia-Goiás, from August 2013 to October 2014, with a group of patients enrolled in the Sistema de Monitoramento dos Radioacidentados (SISRAD) of the Centro de Assistência a Radioacidentados (C.A.R.A.). A total of 102 radioactive patients were divided into two groups: group 1 with 40 and group 2 with 62 participants. A field survey was conducted with a closed and semi-structured questionnaire in which the following contexts were addressed: sociodemographic profile, life habits and personal background. A database was created using the Google Forms application from the Google Web technologies company. The duly collected and stored data were imported and analyzed in the statistical software SPSS, version 21.

**Results:**

The prevalence of SAH reached a total of 25% (12 individuals) of the 48 interviewees, 50% of women (24) and 50% of men (24), of which 22.9% (11) of the radioactivity victims revealed to be smokers.

**Conclusion:**

The prevalence of SAH in the radioactivity victims population is similar to that of the population in general.

## Introduction

In September of 1987 occurred in the state of Goiás, in the city of Goiânia, the radiological accident involving the cesium-137. It was caused by the rupture of a radiotherapy device containing cesium-137 (radioactive isotope), incorrectly handled by lay people and that was abandoned in an inactive medical clinic.^1^

About 112,000 people were involved in this accident, of which 249 were externally or internally contaminated.^1^ The government of Goiás State, through the Municipal Health Department, set up a dedicated service to care for this contaminated population.

Thus, they were categorized in groups I, II and III according to *International Atomic Energy Agency* (IAEA) standards, taking into account classification criteria according to the severity of the cutaneous lesions and the intensity of internal and external contamination.^2,3^ The *Centro de Atendimento aos Radioacidentados* (C.A.R.A.), through the IAEA standards, classifies the radioactivity victims into three groups as follows:

- **Group I (booking each 6 months):** 40 patients with radiodermatitis and/or cytogenetic dosimetry above 0.20 Gy (20 rad) and/or body activity ≥ ½ LIA, corresponding to 1.85 GBq (50 mCi) ;
- **Group II (annual schedule):** 62 patients with cytogenetic dosimetry between 0.05 and 0.20 Gy (5 and 20 rad) and or body activity below ½ LIA; and
- **Group III:** 880 professionals who dealt and deal with contaminated material or patients irradiated or contaminated by cesium-137 and of neighboring population of contamination outbreaks.

The C.A.R.A. is the successor of part of the attributions of the extinct SuLeide (Leide das Neves Superintendence).^2^ This coordinates the referral and counter-referral system of the radioactivity victims and also monitors their health. In addition, it acts in the production of epidemiological data about exposure to ionizing radiation by Cesium-137.^2^

Radiation causes a series of changes in the human body, being of physical, physicochemical, chemical and biological characteristics.^4^ Understanding cellular responses to ionizing radiation is essential for the development of predictive markers useful for assessing human exposure, scarce in the literature.^5^

The threshold dose for circulatory diseases is 0.5 Gy for morbidity and mortality.^4^ Cardiovascular lesions are varied and include accelerated atherosclerosis, pericardial and myocardial fibrosis, conduction abnormalities, and heart valve lesions.^6^ The risk of cardiovascular diseases radiation-related may be correlated to the risks of hypertension and other secondary disorders, such as the risk of atherosclerotic disorders.^6-8^

Systemic Arterial Hypertension (SAH) is considered a chronic, multifactorial degenerative disease whose blood vessel wall pressure values are > 140 mmHg for systolic pressure and > 90 mmHg for diastolic blood pressure.^9^

As a polygenic syndrome, it comprises genetic, environmental, vascular, hormonal, renal and neural aspects.^10^ The control of SAH begins with the detection and continuous observation of blood pressure, prevention of modifiable risk factors and greater access to medicines, Especially, by the unified health system (SUS).^11,12^

The importance of knowing the SAH for the scientific community and for the studied group, is determinant in the systematic follow-up of the victims of the cesium accident for the prevention and monitoring of injuries, since these patients are unique in the world.

It is also relevant to deepen the study because the relationship between hypertension and radioactivity victims patients has been little explored in medical literature. Other studies conducted with radioactivity victims, such as the reflection of psychosocial aspects on the victims of the accident, were well exploited. However, so far there are few publications linking SAH with radioacticity victims. This study aims to contribute with studies that address the primary prevention and early diagnosis for SAH.

The study aimed to know the prevalence of SAH in patients exposed to Césio 137 in an accident in Goiânia-Goiás, registered in the SISRAD of C.A.R.A.

## Methods

this is an epidemiological study of descriptive, observational, cross-sectional design, carried out in Goiânia-Goiás, from August 2013 to November 2014, with patient groups enrolled in C.A.R.A. SISRAD, a unit of the of Health State Department of Goiás.

After the approval of the Ethics and Research Committee, data collection was started, guided by SISRAD, with 102 registered patients referring to groups I and II, respectively 40 and 62 patients. 9 of these died, 8 migrated to other states, 4 migrated out of the country, 2 with not found address and 2 resident in other cities in the state of Goiás and were not found, totaling 25 victims.

However, during the data collection in visits at home in the capital and metropolitan region of Goiânia, 23 patients refused to participate in the study, and it was not possible to locate the address of 3 and 3 were not found after two visits.

In this context, the study was carried out with 48 victims whose SISRAD information used were: name, address and date of birth.

The data collection instrument consisted of the sociodemographic profile, life habits and personal antecedents with structured and semi-structured queries, containing 41 questions.

For the sociodemographic profile, 18 questions were used to characterize the radioactivity victim patient. The remaining 23 questions were divided into life habits and personal antecedents. Concerning life habits, there were questions related to smoking (Fagerstrom test), alcoholism, physical activity and food. As for the personal history, there were questions identifying patients with a previous diagnosis of systemic arterial hypertension and its control with the use of antihypertensives.

The variables were represented by means of descriptive statistics with frequency (absolute and relative) analysis for all participants. Cross-checking between these variables was also performed using statistical software SPSS version 21.

The data was transported to *Google Forms* and stored, being exported to the statistical software SPSS, version 21.

## Results

The study consisted of 48 individuals belonging to groups I and II, according to classification previously mentioned. Of these, 24 (50%) are women and 24 (50%) are men, with a minimum age of 18 and a maximum of 89 years, being the largest number of patients between the ages of 30 and 59 years. The children of individuals in groups I and II were enrolled in these groups and are followed up by C.A.R.A.

About the monthly income, 26 patients (54.2%) receive up to 2 minimum wages; 13 patients (27.1%) have income from 4 to 10 minimum wages with reference to the minimum wage amount of R $ 724.00. Of these, 25 (64.1%) declared as economic activity to be pensioners, as explained in Table 1.

**Table 1:** Sociodemographic characteristics of the 48 participants investigated for Systemic Arterial Hypertension (SAH), radioactivity victims with Césio 137, living in Goiânia-Goiás, Brazil

Characteristics	HYPERTENSE	NON HYPERTENSIVE
Mean age (years): 49 (18 a 89)	61	45
**Age range**		
18-29	01	04
30-39	00	11
40-49	03	05
50-59	03	11
60-69	01	03
70-79	01	02
80-89	03	00
**Gender**		
Male	05	19
Female	07	17
**Get to know SAH normal band**		
Yes	46	----
No	2	----
**Civil status**		
Single	06	-----
Marriage/unmarried union	31	----
Divorced/widowed		-----
**Family income**		
2 minimum wage	26	
2 to 4 minimum wage	08	
4 to 10 minimum wage	13	
> 10 minimum wage	01	

In terms of life habits, 27 patients reported frequent use of alcohol, 12 (44.4%) reported drinking 1 to 2 times a week; 11 (22.9%) patients stated that they are smokers; 42 (87.5%) reported not considering their salt-rich diet; And 39 (81.2%) reported never or rarely practicing physical activity. When asked if they had already been diagnosed with any disease before the accident with Cesium-137, 44 (91.6%) said they did not.

When questioned about having a clinical diagnosis of hypertension, 36 (75%) of the total of interviewees stated that they did not have the diagnosis and 12 (25%) knew they were hypertensive, and of these 7 (29.2%) were female and 5 (20.8%) male, as shown in Table 2.

**Table 2:** Distribution of frequency of interviewees according to gender, estimated monthly income and medical diagnosis of SAH.

	You have medical diagnosis for SAH		Total
	No	Yes	
**Gender**			
Female	17	7	24
	70,8%	29,2%	50%
Male	19	5	24
	79,2%	20,8%	50%
Total	36	12	48
	75,0%	25,0%	100%
Up to 2 minimum wage	19	7	26
	52,8%	58,3%	54,2%
From 2 to 4 minimum wage	7	1	8
	19,4%	8,3%	16,7%
From 4 to 10 minimum wage	9	4	13
	25,0%	33,3%	27,1%
From 10 to 20 minimum wage	1	0	1
	2,8%	0%	2,1%
Total	36	12	48
	75,0%	25,0%	100%

Also in Table 2, the frequency distribution according to the gender of patients who report having a medical diagnosis of SAH with an estimated monthly income is shown. According to the data presented, the income of up to 2 minimum wages corresponds to 7 (58.3%) interviewed, from 2 to 4 minimum wages equals 1 (8.3%) and from 4 to 10 wages minimum is equal to 4 (33.3%).

Table 3 shows the systolic and diastolic blood pressure, in the first and second measurements, according to the classification of the VI Brazilian Guidelines for Hypertension (DBH).

**Table 3:** Frequency distribution of the interviewees according to the blood pressure measurement in the first and second measurements according to the classification of the VI Brazilian Guidelines for Hypertension

Blood Pressure Levels	1^st ^measure		2^nd^ measure	
	Freq.	Percent	Freq.	Percent
Excellent	23	47.92%	24	50.00%
Normal	1	2.08%	4	8.33%
Bordering	7	14.58%	5	10.42%
Hypertension stage 1	10	20.83%	9	18.75%
Hypertension stage 2	6	12.50%	5	10.42%
Hypertension stage 3	1	2.08%	1	2.08%
Total	48	100.00%	48	100.00%

In the first measurement, the optimal classification represents 23 (47.91%) interviewees; The normal classification is equal to 1 (2.08%); The borderline classification is equal to 7 (14.58%). Already the classification stage 1 hypertension represents 10 (20.83%) interviewed; Stage 2 equals 6 (12.5%) and stage 3 equals 1 (2.08%).

In the second measure, the optimal classification represents 24 (50%) interviewees; The normal classification is equal to 4 (8.3%); The borderline classification is equal to 5 (10.4%). The classification of stage 1 hypertension corresponds to 9 (18.7%); Stage 2 corresponds to 5 (10.4%) and stage 3 corresponds to 1 (2.08%).

Considering the clinical diagnosis of hypertension, 15 (31.25%) individuals were identified as hypertensive in the second measurement and 5 (10.42%) presented borderline results.

Table 4 represents the frequency of smoking patients by age group with a predominance of age between 50 and 59 years with 5 (45.5%) smokers.

**Table 4:** Frequency Distribution of interviewees' according to age group and use of tobacco

		You smoke		Total
		No	Yes	
Faixa Etária	18 to 29	5	1	6
		13,5%	9,1%	12,5%
	30 to 39	11	1	12
		29,7%	9,1%	25,0%
	40 to 49	5	2	7
		13,5%	18,2%	14,6%
	50 to 59	9	5	14
		24,3%	45,5%	29,2%
	60 to 69	3	1	4
		8,1%	9,1%	8,3%
	70 e mais	4	1	5
		10,8%	9,1%	10,4%
Total		37	11	48
		100,0%	100,0%	100,0%

Among those who do not smoke, there is a prevalence in the age group of 30 to 39, with 11 (29.7%) smokers, followed by age groups of 50 to 59 years old, with 9 (24.3%).

## Discussion

The results showed that most of the radioactivity victims had no medical diagnosis of SAH. However, there was a prevalence of SAH identified in these subjects of 25%, that is, similar to that of hypertensive individuals in Brazil.

Therefore, it is relevant to study chronic diseases such as hypertension, since its prevalence in populations not exposed to Césio-137 is 28% in Brazil.^12^

It is possible to infer that low income is a socioeconomic factor that interferes in the early diagnosis and control of hypertension, since those with income less than 2 minimum wages have less access to consultations, and less financial condition for the purchase of medications.^16-17^ Several are the determinants for non-adherence to treatment, such as the patient's lack of knowledge about the disease, low socioeconomic status, and high cost of medications.^9^

Another relevant risk factor for SAH in radioactivity was smoking, because smoking causes an acute increase in blood pressure and heart rate, which persists for more than 15 minutes after smoking a cigarette, as a consequence of stimulation of the sympathetic nervous system, at the central level and at the nerve endings.^13^

"The prevalence of smokers was 17.2% of the population aged 15 years or more in 2008, demonstrating the decline that occurred along these 20 years."^14^

A study conducted in the state of Rio Grande do Sul clames that men still smoke more than women, 38% to 29.6%, and smokers with more than 20 cigarettes/day make up the majority: 17.8% of the 33,9%.^11^ These data corroborate with the study presented here, since it reveals that the number of smokers is higher in patients over 50 years and may be influencing the increase of the index of hypertensive in the radioactivity victims.

Researchers from the city of Goiânia recommend the continuation of the studies since the late effects of the radiological accident.^1^ This is because, until the present day, the monitoring reports do not indicate statistically significant data for morbidity and mortality associated with the effects of ionizing radiation, and the somatic effects can be divided into acute or in short-term and late or in long-term effects, depending on the duration of this effects, which is a function of the absorbed dose.^1,4^

The study presented limitations in terms of population and sample. The loss of individuals enrolled as group III in the SISRAD decreased the impact of this study identified as a potential sampling selection bias between groups I and II included in the sample used. Another limitation was the impossibility of reaching individuals of groups II and III, who would have larger samples. It was also not possible to define the causality of hypertension in radioactivity victims patients.

Therefore, the lack of information about cesium-137 on the risk of causing SAH, allows us to affirm that the complications of this disease are irreversible and possibly the level of ionizing radiation has caused long-term changes associated with comorbidities such as hypertension.

### Final considerations

The dissemination of information about cesium-137, concerning the risk of causing hypertension, allows us to affirm that the complications of this disease are significant. Based on this exploratory study, it was not possible to identify that the level of ionizing radiation causes long-term changes associated with comorbidities such as hypertension.

The lack of studies about the health situation of this population, not only in relation to systemic arterial hypertension, but also of other pathologies, especially related to mental health, encourages the development of new researches. This was confirmed by the authors when the data collection instrument was applied, and it was possible to infer to this condition the difficulty found for adherence to this study.

This way, the study concludes that in the population of radioactivity victims the SAH occurs in a similar way to the population in general.
